# Engage? Disengage? Discharge? Ethical approaches to fraught questions

**DOI:** 10.1192/bjb.2025.4

**Published:** 2025-10

**Authors:** Josephine Fielding, Rachel Swain, Jo Emmanuel, Graham Behr

**Affiliations:** 1 Hounslow Psychotherapy Service, West London NHS Trust, London, UK; 2 West London Forensic Services, West London NHS Trust, London, UK; 3 Central and North West London NHS Foundation Trust, London, UK; 4 Westminster Adult Mental Health Services, Central and North West London NHS Foundation Trust, London, UK

**Keywords:** Ethics, consent and capacity, patients/service users, community mental health teams, quality of life

## Abstract

This article presents a framework to assist with the making of often challenging decisions about engagement and disengagement with patients across mental health services. The framework is based on Beauchamp & Childress’s four principles of clinical ethics. We pose practical questions, illustrated by a clinical vignette, around these four principles in order to aid implementation of ethics-based decision-making. The framework is useful in both complex and seemingly straightforward issues. It can be used as a means of communicating what are often controversial decisions to fellow clinicians and patients.

Decisions regarding engagement with mental healthcare can be unusually fraught and can be a source of distress for patients and professionals alike. As clinicians, we are all familiar with anxiety-inducing decisions about the appropriate level of engagement with a patient, be that someone who is not engaging with treatment or someone who is engaging with care but where there is disagreement on whether that care is helpful for them. Teams often persist in offering care even when there does not seem to be meaningful benefit. Finding the balance between assertive outreach, coercion and knowing when to step back is difficult.

Healthcare providers, as organisations and as individuals, navigate changing expectations informed by the resources and culture of the society in which they are embedded, and these may fluctuate over time.

Mental health providers are particularly familiar with the quandary of caring for people who do not want to be cared for. Conversely, organisations may be expected to provide care to mitigate the anxiety associated with perceived risk, even where there is no evidence of benefit. In Western culture there is a sense that the responsibility for mitigation of such things as self-harm, suicide and threat or harm to others rests with mental health services, and these outcomes are often attributed by society to deficits of care. Szasz^1^ and Foucault^2^ were early critics of the role of psychiatry, and recent commentators such as Timimi^3^ point to the lack of consensus regarding the reach and scope of mental health services and where responsibility for responding to emotional distress lies. Societal concerns about risk,^4^ legal routes enabling coercive treatments and fear of blame can all get in the way of meaningful dialogue with patients about what would be truly helpful for them. Decisions about engagement and disengagement are often made without reference to these wider issues, which can lead to conflicting views based on differing underlying and unexamined assumptions. These situations can also be particularly emotionally charged, so teams need help to understand and reflect on their emotional responses and how these affect decision-making in both helpful and unhelpful ways.

Disengagement from mental health services is a significant problem with serious consequences and poorer outcomes.^5^ Consequently, there are particular reasons why mental health services may wish to be more assertive in their outreach. In mental healthcare, difficulties in engagement can be due to the very nature of the presenting illness or to the fact that patients may face more structural barriers to accessing healthcare. Patients may experience significant internal conflict in accessing treatment, including different understandings of illness, shame, self-stigma, fear of consequences for employment and negative previous experience of services. Those accessing mental health services are also more likely to have insecure attachment styles, which affect treatment engagement.^6,7^ All of these factors should be woven into judgements made when considering the principles discussed below.

## Aims

We propose using a framework based on Beauchamp & Childress’s four principles of clinical ethics,^8–10^ showing how they apply to decisions regarding engagement and disengagement. We will then set out how they translate into questions that practitioners can use to guide their exploration of these issues, drawing in part from our experience on clinical ethics committees. We do not claim to provide comprehensive guidance; rather, we wish to suggest a starting point which teams can use to promote more nuanced and ethically informed conversations.

We use the following fictitious case vignette to highlight the use of some of these principles.

### Case vignette – Ella

Here we present a fictitious case vignette. Ella has a diagnosis of emotionally unstable personality disorder. She frequently presents to the accident and emergency department (A&E) with self-harm. During hospital admissions, there are frequent instances of assaulting others when her needs are not met promptly.

She engages erratically in the community and has not been able to benefit from psychological therapy. She does ring the crisis team out of hours when in distress or presents to A&E asking for help.

Following a significant overdose, for which she refuses treatment, she is detained under the Mental Health Act and receives treatment under duress for both her physical and mental health.

After several weeks on the ward, opinion is divided about whether she should remain under services, be they in-patient or in the community.

## A four principles approach to engagement

The four principles approach uses the principles of autonomy, beneficence, non-maleficence and justice to build a framework for ethical decisions. We explore these in more detail below, relating them to Ella’s case in the text and in Table 1.

**Table 1:** The four principles of clinical ethics in relation to Ella’s case

Principle	Definition	In Ella’s case
Autonomy	The right of an individual to, when able, choose freely	This rests on careful exploration of whether Ella has capacity, or not, to engage responsibly with treatment
Beneficence	To act for the benefit of the patient	The perceived short-term benefit of staying in the safety of the ward environment needs to be balanced against the potential longer-term benefit of enhancing her sense of agency through discharge to the community. Both options also incur potential harm to Ella or others – essentially requiring weighing up these conflicting approaches with respect to benefit and harm
Non-maleficence	To minimise harm	
Justice	Fair, equitable and appropriate distribution of healthcare resources	Consideration of the resources appropriate to meet Ella’s complex needs (e.g. through extended in-patient stay – a high-cost intervention) balanced against whether, in the absence of clear benefit, this use of resources is disproportionate

### Autonomy

Autonomy, the principle underpinning self-determination, allows those who are competent to make their own informed decisions. Beauchamp & Childress emphasise, however, that this right is underpinned by capability and that the principle of autonomy is not absolute.

Capacity is used as a proxy marker for this capability, but judgement of this is inherently subjective. Our personal values cannot help but influence our views and thus how we interpret other people’s decisions as correct or unwise.^11^ Lived experience frames patients’ weighing and judging of decisions, and they may have a different conception of what ‘improved’ or ‘well’ means compared with health professionals.^12^ Where patients are making what are perceived as unusual or potentially harmful decisions, this can in itself lead clinicians to doubt their capacity. Conversely, where patients agree with clinicians, this sways professionals to judge them as capacitous.^12^

Beale^13^ considers the idea of superficial assessments concerned with capacity as a rationalisation for abrogation of clinician responsibility and inadequate healthcare resources, and cautions against this. This highlights the importance of being vigilant against our own biases and countertransference responses when assessing capacity. If we assumed complete responsibility for all those presenting with acts of self-harm, however, this would lead to unconscionably paternalistic practice. Despite their shortcomings, capacity assessments are the best tool we have to determine patients’ ability to make autonomous choices, and decisions on behalf of others can only be justified where autonomy is lacking.

Respecting a patient’s autonomy can mean giving people ‘the dignity to fail’.^14^ Not getting things right and making unwise decisions are a part of human development and growth, and allowing them could be considered respectful of autonomy and self-determination. This may be equally true of assertive outreach that reverses a psychosis. Thus, engaging with and disengaging with patients are potentially both pathways towards maximising autonomy.

In terms of autonomy, there are two key issues for clinicians to consider in respect of engagement, whether that be closing a case, discharging from a ward, assertive outreach or coercion. The first is whether the person has autonomy of thought or action in their ability to engage with care. Can they appreciate the need, judge the pros and cons and have they the means to act on that judgement? Mental health problems are associated with risky behaviour; thus, the terms of both engagement and non-engagement need to be informed by a second question, namely whether that individual should be regarded as capable of taking responsibility for that behaviour.

In respect of Ella, the in-patient clinicians may come to the view that she is too aroused to be responsible either for her decision to remain on the ward or for her actions that endanger her. This may lead to a decision that she lacks autonomy and therefore to the decision not to discharge her or even to consider detaining her and using coercive treatment. Conversely, they may feel that she is capable of engaging responsibly with community services as she has shown the ability to seek help when needed. The community team may similarly decide not to engage her assertively, because she has sufficient agency to seek help as needed and that she is capable of the decision to engage constructively or not and to manage her own safety. However, decisions about discharge must also consider the remaining three principles.

The principles of beneficence and non-maleficence clearly need to be considered together and may usefully be thought of as ‘net benefit’,^15^ but we describe them here independently.

### Beneficence

Beneficence refers to the likely benefit of an intervention, based on the evidence of the likely benefit of that intervention. It can be considered as promoting patients’ overall good and well-being. This is less straightforward than first appears however. Beneficence is both time- and patient-specific, consequences of interventions can be hard to predict and benefit can at times arise from not providing care, which may be harder for clinicians to keep in mind.

Both evidence-based medicine and the patient’s previous response to treatment can assist us in predicting benefit. However, judging the likelihood of benefit is particularly difficult in psychiatry because of the complex interplay of personal (individual) factors affecting response to treatment (unlike the group characteristics underpinning much treatment evidence), including culture, social circumstances, personal history and relationship with services. Whether someone is able to make use of a treatment offered, for example psychotherapy or substance misuse treatment, will depend heavily on their motivation and openness to experiencing challenge and difficult feelings in the service of change. The value of an intervention could therefore be very different at different points in the patient’s journey.

A further important consideration is whether there are any benefits in not providing care or in respecting the person’s wishes to disengage from care. As Corrigan et al^16^ argue, treating the patient as a rational agent may be beneficial in the longer term in promoting the development of their own agency and self-determination.

Attention needs to be paid also to the scope of beneficence.^15^ We have duties to care not only about our patients but their families and carers, the communities around them and wider society, as well as the welfare of ourselves and our colleagues as the providers of care.

In respect of Ella, the clear benefit of staying on the ward might be the containment and oversight that prevents self-harm, as well as the potential ability to begin more durable therapeutic interventions. We may also be sparing her and her family anxiety in the short term. Conversely, we must consider the lack of benefit of previous hospital stays, the diminishing of her own sense of agency and responsibility, as well as the aggression and violence endured by the nurses and other patients.

Ella’s case illustrates the importance of scrutinising proportionality: a prolonged hospital stay does not appear to be benefiting her. It could be argued that community care might shift the locus of control to Ella and enhance her long-term prospects to the degree that warrants short-term risk-taking.

### Non-maleficence

Like beneficence, ‘not doing harm’ seems at first glance straightforward (is the proposed intervention likely to cause harm?) – but is actually often hard to identify. Non-maleficence also needs to be considered through a wider lens than harm to the patient, for example considering the harms of Ella’s antisocial behaviour to others on the ward.

Short-term harm may be offset by long-term benefit, for example deprivation of liberty for treatment through detention or community treatment orders (which are typically perceived by patients as ‘harmful’). The availability, in many countries, of legal routes for enforced treatment allows clinicians to act decisively to prevent harm. However, the possible harms of coercive treatment have been well established,^17^ including the potential for damage to patients’ long-term relationship with services and trust in professionals. Various types of pressure that fall short of frank coercion can also have an impact on people’s experiences of care.^18^ Even in the absence of overt coercion, relationships with services in which there is no room for negotiation may reduce people’s sense of agency and hinder potential for personal growth.^19^

It is easy to underestimate the potential for harm from well-intentioned interventions. Roe & Davidson^12^ recognised the danger of minimising the potential risks of a treatment in favour of exaggerating potential gains. Linden^20^ also highlights the potential for adverse effects from non-pharmacological interventions such as psychotherapy.

It cannot be automatically assumed that illness is always a ‘greater evil’ than the effects of its treatment, and patients who do not engage may be communicating that they find the symptoms themselves more tolerable than treatment. Furthermore, the resolution of symptoms is not always beneficial, for example delusional reality may be more bearable than loss, loneliness or feelings of insignificance. Chase et al^21^ have also described the harm or challenge to core values and identity when people transition from ‘independent agent to psychiatric patient’.

Disengagement from services is frequently thought of as a risk in and of itself, which it clearly is not, unless known to be a marker of deterioration for that particular person. The difficulty of predicting risk, especially low-frequency outcomes, has been well described,^22^ but fear of risk can lead to overly paternalistic practice, including assertively seeking to ‘know’ about people when they are not engaging with us.

It can be tempting for clinicians to consider the more overt and immediate risks and prioritise preventing those, but this risks neglecting more subtle and long-term harms.

### Justice

Justice can incorporate multiple dimensions in terms of ethical decision-making, including fair distribution of scarce resources, respect for people’s rights and respect for morally acceptable laws.^15^ We will focus on the first of these, distributive justice.

At an individual level, giving equal access to those in equal need (horizontal equity) and treating them as moral equals is one of the founding principles of the UK’s National Health Service. Patients are not all equal, however, and vertical equity, the notion that we should be treating unequals unequally, comes into play. Ella’s complex needs, for example, might require greater support, necessitating an unequal allocation of resources. It can be difficult to judge levels of relative need in planning and delivering services. We must be mindful of how our own unconscious biases, issues of countertransference, patient or family demands or subjective ideas about who is ‘deserving’, can cloud our judgement about who is in greatest need.

Using copious resources for some patients, without curtailing those available to other patients, brings us face to face with the reality of the ‘bottomless pit’ of wants.^23^ It is unachievable in a healthcare system with finite resources. The idea of welfare maximisation means we are responsible for considering how to maximise the benefit to the greatest number of people given finite resources. Although Doyal^24^ speaks of everyone having the right to good healthcare as a means to being able to flourish in a society, he notes that we are not, in contrast, entitled to good health. There will be instances where offering care is not substantially beneficial, in which case disengagement of services could be considered the socially moral thing to do. This might be the case for aspects of Ella’s care, such as her ongoing admission, where limited evidence of clinical benefit may justify making the bed available to another patient instead.

Making decisions about engagement and disengagement demands respect for fair allocation of resources and an honest assessment of cost–benefit, be that clinicians’ time, priority on waiting lists, bed space or medication.

We do not work in isolation, however, but in an ecosystem of health and societal provisions. Rationing resources in one aspect of society is likely to have a knock-on effect in another. For example, disengagement of a mental health team could increase contact with police or ambulance services. The variety of different lenses through which distributive justice can be viewed are often competing. As Gillon^15^ comments based on Calabresi & Bobbitt,^25^ we should not therefore be surprised, when trying to juggle these conflicting ideas, that not all accounts are satisfactorily resolved simultaneously.

In making decisions about engagement and disengagement in the context of finite resources, it may be useful to ask the following questions of ourselves. These questions will help us address horizontal equity, vertical equity and welfare maximisation.

1. All things being equal, is everybody getting equal access?
2. How great is the need in this person/group in the context of the others eligible?
3. Is the benefit proportionate to the resources utilised?

## Conclusion

The four principles of clinical ethics provide an overarching framework. They are not intended as a set of rules to follow but a starting point for more nuanced discussion in a more structured way. Although formal ethics case conferences – either using clinical ethics committees or *ad hoc* discussions with people with relevant expertise – can bring great value, our hope is that this explanation is helpful for all clinicians to use within their own teams. Applying this is not always straightforward – indeed these are principles underpinning a framework and not in any way intended as a set of rules to follow. To that end it may help to consider the following key questions.

1. Does the person have the capacity to decide whether to engage with the care offered?
2. Do they have capacity to take responsibility for the risks they are taking/exposed to?
3. What is the net benefit of offering (or withholding/withdrawing) care at this time? Is the benefit proportionate to any harm?
4. Is this a fair use of resources?
  - How much in need is the person in the context of the others eligible for care?
  - Is the benefit proportionate to the resources utilised?

These questions may give rise to conflicting tensions, but will provide an explicit moral rationale to underpin decision-making and, crucially, to communicate these decisions and their reasoning to patients and to colleagues. We suggest that this approach makes decision-making more robust and free from the baggage of excessive risk aversion and the constraints of diagnostic labels. It helps minimise value-laden judgements and avoids privileging intuition, random group consensus or deference to authority in making tough decisions.

Although ethics is often invoked in more troubling scenarios, we believe that this approach is of wider benefit to patients, including those whose care seldom has a light shone on it.

## About the authors

**Josephine Fielding** is a consultant psychiatrist in psychotherapy with West London NHS Trust, UK. **Rachel Swain** is a consultant forensic psychiatrist with West London NHS Trust, UK. **Jo Emmanuel** is an honorary consultant psychiatrist with Central and North West London NHS Foundation Trust, UK. **Graham Behr** is a consultant psychiatrist, Central and North West London NHS Foundation NHS Trust, UK.

## Data Availability

Data availability is not applicable to this article as no new data were created or analysed in this study.
